# *In vitro* and *in vivo* evaluation of periosteum-derived cells and iPSC-derived chondrocytes encapsulated in GelMA for osteochondral tissue engineering

**DOI:** 10.3389/fbioe.2024.1386692

**Published:** 2024-04-11

**Authors:** Hannah Agten, Inge Van Hoven, Jasper Van Hoorick, Sandra Van Vlierberghe, Frank P. Luyten, Veerle Bloemen

**Affiliations:** ^1^ Department of Materials Engineering, Surface and Interface Engineered Materials (SIEM), Group T Leuven Campus, KU Leuven, Leuven, Belgium; ^2^ Skeletal Biology and Engineering Research Center, KU Leuven, Leuven, Belgium; ^3^ Prometheus, Division of Skeletal Tissue Engineering, KU Leuven, Leuven, Belgium; ^4^ BIO INX BV, Zwijnaarde, Belgium; ^5^ Polymer Chemistry and Biomaterials Group, Centre of Macromolecular Chemistry, Ghent University, Ghent, Belgium

**Keywords:** tissue engineering, cartilage, osteochondral, induced pluripotent stem cell-derived chondrocyte, serum-free

## Abstract

Osteochondral defects are deep joint surface lesions that affect the articular cartilage and the underlying subchondral bone. In the current study, a tissue engineering approach encompassing individual cells encapsulated in a biocompatible hydrogel is explored *in vitro* and *in vivo*. Cell-laden hydrogels containing either human periosteum-derived progenitor cells (PDCs) or human induced pluripotent stem cell (iPSC)-derived chondrocytes encapsulated in gelatin methacryloyl (GelMA) were evaluated for their potential to regenerate the subchondral mineralized bone and the articular cartilage on the joint surface, respectively. PDCs are easily isolated and expanded progenitor cells that are capable of generating mineralized cartilage and bone tissue *in vivo* via endochondral ossification. iPSC-derived chondrocytes are an unlimited source of stable and highly metabolically active chondrocytes. Cell-laden hydrogel constructs were cultured for up to 28 days in a serum-free chemically defined chondrogenic medium. On day 1 and day 21 of the differentiation period, the cell-laden constructs were implanted subcutaneously in nude mice to evaluate ectopic tissue formation 4 weeks post-implantation. Taken together, the data suggest that iPSC-derived chondrocytes encapsulated in GelMA can generate hyaline cartilage-like tissue constructs with different levels of maturity, while using periosteum-derived cells in the same construct type generates mineralized tissue and cortical bone *in vivo*. Therefore, the aforementioned cell-laden hydrogels can be an important part of a multi-component strategy for the manufacturing of an osteochondral implant.

## 1 Introduction

Osteochondral (OC) defects are severe joint surface defects that progress past the articular cartilage and extend into the subchondral bone (Slattery and Kweon, 2018). These defects are more complex than chondral defects due to the multiple tissue types involved (Atala et al., 2012; Jacob et al., 2020). In contrast to articular cartilage, subchondral tissue is highly vascularized and has cellular repair mechanisms to facilitate tissue remodeling and regeneration (Zhou et al., 2020). Due to the access to the bone marrow space, progenitor cells can be recruited to the defect site, where they will proliferate, differentiate, and produce the extracellular matrix. New subchondral bone is formed, as well as cartilage repair tissue; however, this tissue is high in collagen type I, which is associated with the mechanically inferior fibrocartilage as opposed to the hyaline cartilage (Nakamura et al., 2020). Fibrous cartilage will protect the joint surface initially but cannot functionally resist the forces exerted on a load-bearing joint, eventually leading to the degeneration of the articular surface (Madry et al., 2010). Therefore, deep osteochondral defects usually require a therapeutic intervention. Techniques used for chondral lesions such as autologous chondrocyte implantation (ACI) do not suffice for these defects since the interface with the damaged subchondral bone should also be addressed (Yang et al., 2017a). The most used strategy for OC defects is osteochondral grafting (defect <2 cm^2^) or the use of multiple cylindrical grafts in a technique referred to as mosaicplasty (defect > 2 cm^2^) (Ondrésik et al., 2018). Healthy tissue is taken from a non-load-bearing region of the knee or from a donor and transplanted into the damaged area. The challenges of this technique lie in 1) the availability of healthy donor tissue, 2) problems at the donor site, and 3) congruency and integration of the plugs at the reception site (Yang et al., 2017a; Jacob et al., 2020).

Tissue engineering strategies aim to regenerate damaged tissues to sustainably relieve symptoms (Ondrésik et al., 2018). Developing a customized tissue engineered plug addresses problems with graft availability and donor site morbidity. Congruency issues and integration problems at the edges of the plug could be targeted by clever implant design and construct fabrication processes (Yang et al., 2017a).

The osteochondral tissue has a unique structure, with biochemical and biomechanical properties that change from top (articular cartilage) to bottom (subchondral bone). The transition between the articular cartilage and subchondral bone is made by a layer of mineralized cartilage.

For the generation of articular cartilage, obtaining adequate amounts of healthy chondrocytes is challenging. First, these chondrocytes can only be harvested from healthy tissue and not from a diseased joint area. Second, from a healthy joint region, only small biopsies can be taken without initiating secondary damage (Foldager, 2013). Additionally, the *in vitro* expansion of chondrocytes faces issues with dedifferentiation (Ma et al., 2013). Therefore, researchers considered the use of stem cells or progenitors that can be either obtained in larger amount or can be expanded *in vitro* prior to differentiation by using stimulating factors (Canadas et al., 2018). Recently, the use of induced pluripotent stem cells (iPSCs) has gained attention since these pluripotent stem cells can be generated from adult, somatic cells with minimally invasive harvesting. Additionally, these cells display low immunogenicity, which is relevant for allogeneic strategies (Yang et al., 2017a; Ondrésik et al., 2018; Castro-Viñuelas et al., 2020). Mesenchymal stem cells (MSCs) are multipotent adult stem cells that can be derived from various sources in the body such as bone marrow, adipose tissue, and synovial fluid. These cells can be harvested in larger amounts, are easily expanded *in vitro*, and can be differentiated toward chondrogenesis and osteogenesis (Canadas et al., 2018; Ondrésik et al., 2018; Jacob et al., 2020). However, it must be noted that their proliferation and differentiation potential is donor- and source-dependent and varies with age (Kim et al., 2012). In addition, MSC-derived chondrocytes tend to produce fibrocartilage or hypertrophic cartilage that may mineralize and undergo endochondral ossification, thus forming bone eventually (Zhou et al., 2020). The latter is not relevant for articular cartilage regeneration, but it can be relevant for deep joint defects, where the subchondral bone is also affected. It is even postulated that including a calcified layer in a tissue engineered cartilage implant enhances the performance of the construct by increased anchoring with the subchondral bone (St-Pierre et al., 2012).

The progenitor cell source we propose to prepare the bridge between the articular cartilage produced by chondrocytes and the subchondral bone at the bottom of the defect is a population derived from the periosteum. Human periosteum-derived cells (hPDCs) are skeletal progenitor cells that not only play a crucial role in bone development and growth but also in bone fracture healing (Roberts et al., 2015). hPDCs in a scaffold-free setup form mineralized cartilage and bone (Freitas Mendes et al., 2016; Mendes et al., 2018; Nilsson Hall et al., 2020).

Although some scaffold-free approaches have yielded promising results, there are possible advantages of incorporating a supportive matrix into a tissue engineered construct. In particular, hydrogel matrices are suitable for soft and hard tissue regeneration (Yang et al., 2017a). Hydrogels support chondrogenic differentiation amongst others by maintaining the spherical morphology, and they stimulate the integration of tissue engineered constructs with the surrounding tissue (Moreira Teixeira et al., 2014). Moreover, cell-laden hydrogel constructs allow controlled cell distribution, and they are suitable for use in various processing techniques, including automated fabrication, which facilitates more patient-customized implants, adding to congruency and integration (Moreira Teixeira et al., 2014; Yang et al., 2017a; Rajabi et al., 2021). Gelatin methacryloyl (GelMA) is a semi-synthetic hydrogel based on hydrolyzed collagen. GelMA combines a natural polymer with properties similar to those of the native extracellular matrix, such as RGD sequences and matrix metalloproteinase (MMP) target sites, ensuring biocompatibility and biodegradability, with the advantages of a synthetic hydrogel through the MA functionalization, allowing tunability of its mechanical properties (Van Den Bulcke et al., 2000; Klotz et al., 2016; Yang et al., 2017a; Van Hoorick et al., 2017).

Our hypothesis is that the combination of a biomimetic and processable hydrogel matrix and periosteum-derived cells will generate transient cartilage with the potential to mineralize or undergo endochondral ossification *in vivo*. In this study, we aim to show the generation of transient cartilage and stable cartilage using the same chemically defined, serum-free medium formulation. With this approach, both cell-laden hydrogels could be readily combined in a multilayered construct for osteochondral regeneration.

## 2 Materials and methods

### 2.1 Cell culture

Periosteum-derived cells were isolated from biopsies of four donors and pooled and cryo-preserved, as previously described (Eyckmans et al., 2010). A cell pool was used to account for matrix formation variability among the donors. The donors were matched according to age group (age 10–17 years) and similar cumulative population doublings. The use of these biopsies was approved by the Ethics Committee Research UZ/KU Leuven (ML7861). After thawing, the cells were expanded in Dulbecco’s modified Eagle’s medium (DMEM) GlutaMAX™ with 1 µM pyruvate (Gibco, United States) supplemented with 1% antibiotic/antimycotic solution (Gibco, United States) and 10% fetal bovine serum (FBS; Biowest, FR). Upon confluency, the cells were detached using TrypLE Express (Gibco, United States) for counting with trypan blue before passaging or harvesting.

iPSC-derived chondrocytes were obtained by the differentiation of CY2 human iPSCs (Rutgers University Cell and DNA Repository, United States), as described elsewhere (Yamashita et al., 2015). In brief, the cells were expanded on SNL feeder cells treated with mitomycin C (Sigma, DE) in a human embryonic stem cell (hESC) medium consisting of DMEM-F12 (Gibco) supplemented with 1% sodium pyruvate, 20% knockout serum replacement (Invitrogen), 2 mM GlutaMAX (Gibco), 1% non-essential amino acids (NEAAs; Gibco), 0.1 mM β-mercaptoethanol (Sigma), 50 U and 50 mg/mL penicillin/streptomycin (Pen/Strep; Invitrogen), and 10 ng/mL human basic fibroblast growth factor (hbFGF; PeproTech). After sufficient expansion, the iPSCs were transferred to Matrigel-coated well plates and cultured in Essential 8 Medium (Gibco). Next, they were cultured in Essential 6 Medium (Gibco) supplemented with 8 µM CHIR99021 (GSK3β inhibitor; Axon Medchem), 50 U and 50 mg/mL Pen/Strep, and 20 ng/mL hbFGF for 36 h of mesoderm induction, followed by another 36 h in the same medium supplemented with 50 U and 50 mg/mL Pen/Strep, 8 ng/mL hbFGF, and 1 µM retinoic acid. Next, the formation of cartilage-like nodules was stimulated by culturing the cells in a chondrogenic medium consisting of DMEM GlutaMAX™ with 1 mM pyruvate supplemented with 1% FBS, 1% L-glutamine (Thermo Fisher, United States), 1% MEM Non-Essential Amino Acids Solution (100×) (Gibco, United States), 0.05 ng mL^-1^ ascorbic acid L-variant (Sigma, DE), 50 U mL^-1^ penicillin–streptomycin (Gibco, United States), 82.5 µM β-mercaptoethanol (Gibco, United States), and 1% insulin–transferrin–selenium–ethanolamine (ITS-X) (100×) (Gibco, United States). Growth factors were added at a concentration of 10 ng mL^-1^: transforming growth factor β1 (TGF-β1; PeproTech, United Kingdom), growth and differentiation factor 5 (GDF-5; PeproTech, United Kingdom), bone morphogenetic protein 2 (BMP-2; PeproTech, United Kingdom), and hbFGF (PeproTech, United Kingdom) for 2 weeks. The nodules were then detached and cultured in suspension for 9 weeks in the same chondrogenic medium without hbFGF. Single iPSC-derived chondrocytes were released from the cartilage-like nodules using two enzymatic digestion steps. First, 2 mg mL^-1^ pronase solution (60 mg, ∼7.0 U mg^-1^, Roche, CH) in PBS +1% antibiotic/antimycotic solution (100×) (AA, Gibco, United States) (30 mL) was filtered (0.22 µm) and applied to the nodules for 25 min whilst rotating at 37°C. After removal of this solution, 1.5 mg mL^-1^ collagenase B solution (45 mg, >0.15 U mg^-1^, Roche, CH) in DMEM-F12 (Gibco, United States) + 1% AA (30 mL) was also sterile-filtered, applied, and incubated whilst rotating at 37°C until full digestion. The cells were passed through a cell strainer (70 µm) to remove matrix debris, centrifuged (10 min, 1,300 rpm), and resuspended in medium for counting with trypan blue.

### 2.2 Construct fabrication

GEL‐MA INX X210 (BIO INX) containing a photoinitiator (Li‐TPO, 0.6 mM [final concentration]) was dissolved at 20% (wt/vol) in sterile PBS at 40°C. A suspension of hPDCs or iPSC-derived chondrocytes in PBS was added to the hydrogel precursor solution to obtain a final GelMA concentration of 10% (w/v) and a cell density of 2 × 10^7^ cells mL^−1^. The cell-laden hydrogels were transferred to custom-made PTFE molds (as done by Loessner et al.), cross-linked with UV light (365 nm, UVP CL-1000) for 10 min and cut into 4 × 4 × 2 mm constructs (Loessner et al., 2016). As positive controls, micromasses consisting of 20 µL of cell suspension with the same density as the cell-laden hydrogels (20 × 10^7^ cells mL^−1^) were seeded into 24-well plates and incubated for 3 h before adding the medium, as described earlier (De Bari et al., 2001). Negative control samples for the *in vivo* studies were GelMA hydrogel constructs without cells, and for the mechanical characterization, cell-laden GelMA constructs cultured in a conventional medium were used as negative controls.

### 2.3 *In vitro* differentiation

Cell-laden GelMA constructs and micromass controls were cultured in 24-well suspension plates (CELLSTAR) in a chemically defined, serum-free differentiation medium consisting of low-glucose DMEM (Gibco) supplemented with 1 mM ascorbate-2-phosphate (Sigma-Aldrich), 100 nM dexamethasone (Sigma), 40 μg/mL proline (Sigma), and ITS + Premix Universal Culture Supplement (BD Biosciences) and growth factors: 10 ng/mL TGF-β1, 0.2 ng/mL fibroblast growth factor 2 (FGF-2; PeproTech), 100 ng/mL BMP-2 (InductOs), 1 ng/mL BMP-6 (PeproTech), and 100 ng/mL GDF-5 (PeproTech), as described by Mendes et al. (Moreira Teixeira et al., 2014). Negative control samples were cultured in a conventional medium consisting of DMEM supplemented with 10% FBS and 1% AA. Samples were cultured for up to 28 days at 37 °C, 5% CO_2_, and 95% RH, and the medium was refreshed every 2 days.

### 2.4 *In vivo* ectopic evaluation

Cell-laden or cell-free constructs (n = 4) were implanted in the subcutaneous pockets on the back of 8–11-week-old female nude mice (Janvier, Rj:NMRInu/nu) after being cultured *in vitro* for 1 day, i.e., unprimed constructs, or for 21 days, i.e., primed constructs (six animals, four constructs per animal, randomized). Under general anesthesia (5 mg/kg xylazine and ketamine 80 mh/kg, intraperitoneal injection), an incision was made on the back of the mice after disinfection of the skin. Four dorsal pockets were created, two at the shoulder region and two at the back region. Samples were washed in PBS and placed in the pockets using a spatula. The skin was closed using surgical staples, and analgesia (0.06 mg/kg buprenorphine) was administered subcutaneously every 12 h for 3 days. The animals were allowed to recover solitarily and placed in groups of four afterward. The animals were euthanized after 4 weeks, and all constructs were explanted and fixed in 4% paraformaldehyde (PFA) (1 mL) for subsequent histological evaluation. The animal experiments were approved by the Ethical Committee for Animal Experiments, KU Leuven (ECD, project P127-2016).

### 2.5 Viability assay

On days 1, 4, and 7 of the *in vitro* culture, cell-laden constructs (n = 3 per condition) were washed twice with PBS (1 mL) and incubated in a LIVE/DEAD™ Viability/Cytotoxicity staining solution (2 µM calcein acetoxymethyl ester and 4 µM ethidium-homodimer-1 in PBS, 1 mL) (Invitrogen, United States) for 30 min at 37 °C, 5% CO_2_, and 95% RH. After washing two more times with PBS (1 mL), the constructs were imaged using an inverted fluorescence microscope (Olympus IX83).

### 2.6 Nucleic acid extraction

On days 1, 14, 21, and 28 of the *in vitro* culture, cell-laden constructs and micromass controls were washed twice with PBS, snap-frozen in liquid nitrogen, and stored at −80°C. The frozen samples were then transferred to lysing tubes (CK-28R, Bertin Instruments, FR) containing 400 µL of buffer RLT (QIAGEN, NL) supplemented with 1% (v/v) β-mercaptoethanol (Sigma, DE). The samples were homogenized using the Precellys homogenizer (4°C, 3 cycles of 15 s at 8,800 rpm with 10-s intervals in between) (Bertin Instruments, FR). The homogenized samples were centrifuged at 18,000 rpm for 10 min at 4°C to sediment debris. The supernatant was used for DNA quantification and RNA extraction.

The supernatant of homogenized samples was diluted to 1:10 in Milli-Q water for DNA quantification to decrease the interference of the lysis buffer. DNA content was quantified using the Qubit™ dsDNA HS Assay Kit (Invitrogen, United States) according to the manufacturer’s protocol.

RNA was extracted from the supernatant of the homogenized samples using an RNeasy Mini extraction kit (QIAGEN, NL) according to the manufacturer’s protocol, and the isolated RNA was quantified using a NanoDrop 2000 spectrophotometer (Thermo Scientific, United States).

### 2.7 RT-qPCR

The total RNA isolated was transcribed to copy DNA (cDNA) using a PrimeScript RT Reagent Kit (Takara Bio, JPN), which was used for quantitative polymerase chain reaction (qPCR) with SYBR Green (Life Technologies, United States). All output was normalized to the housekeeping gene hypoxanthine phosphoribosyltransferase 1 (HPRT1). Gene expression was analyzed using the 2^−ΔΔCT^ method, as described by Livak and Schmittgen (2001) Primer sequences are listed in Table 1.

**TABLE 1:** Primer sequences used for qPCR.

Gene	Forward primer (5′–3′)	Reverse primer (5′–3′)
HPRT1	TGA​GGA​TTT​GGA​AAG​GGT​GT	GAG​CAC​ACA​GAG​GGC​TAC​AA
SOX9	TGG​AGA​CTT​CTG​AAC​GAG​AGC	CGT​TCT​TCA​CCG​ACT​TCC​TC
ACAN	GTCTCACTGCCCAACTAC	GGA​ACA​CGA​TGC​CTT​TCA​C
COL2A1	GGC​TTC​CAT​TTC​AGC​TAT​GG	AGC​TGC​TTC​GTC​CAG​ATA​GC
COL1A1	CAC​GAA​GAC​ATC​CCA​CCA​AT	AGA​TCA​CGT​CAT​CGC​ACA​AC
COL10A1	ACG​ATA​CCA​AAT​GCC​CAC​AG	GTG​GAC​CAG​GAG​TAC​CTT​GC
IHH	AAC​TCG​CTG​GCT​ATC​TCG​GT	GCC​CTC​ATA​ATG​CAG​GGA​CT
CNMD	TCA​GCA​GGA​AGG​GGA​AAG​CA	GAT​GAC​TCT​GCA​GGC​CGA​AC
RUNX2	CGC​ATT​CCT​CAT​CCC​AGT​AT	GCC​TGG​GGT​CTG​TAA​TCT​GA
OSX	AGT​GAC​CTT​TCA​GCC​TCC​AA	GGG​AAA​AGG​GAG​GGT​AAT​CA
ALP	GTG​CAC​CAT​GAT​TTC​ACC​ATT​CTT	TGT​CTC​TTG​CGC​TTG​GTC​TC
OCN	GTG​CAG​CCT​TTG​TGT​CCA​A	GCT​CAC​ACA​CCT​CCC​TCC​T

### 2.8 Histology and immunohistochemistry

On days 1, 14, 21, and 28 of the *in vitro* culture, cell-laden constructs and micromasses were washed twice in PBS (1 mL) and fixed in 4% PFA (1 mL) for 1 h at room temperature, washed twice in PBS, and stored at 4°C before staining. Explants from the ectopic assay were washed and fixed the same way, but after micro-CT scanning, they were decalcified in 0.5 M ethylenediamine tetraacetic acid (EDTA; pH 7.5) for 10 days at 4 °C. The samples were dehydrated by incubation in an ethanol solution series with concentrations of 50%, 70%, and 95% (v/v), embedded in paraffin, and sectioned into 5-µm sections using a microtome. The sections were deparaffinized in Histo-Clear (National Diagnostics, United States), dehydrated in methanol, and rehydrated in deionized water prior to all staining procedures. For Alcian Blue staining, the samples were stained with freshly filtered 0.5% Alcian Blue solution (pH 1), rinsed with deionized water, counterstained with 0.1% Nuclear Fast Red solution (Sigma, DE), and rinsed again with deionized water. For Safranin O staining, the samples were counterstained with 1% hematoxylin solution (Merck, DE), rinsed with tap water, and dipped in acid alcohol (1% HCl in 10% ethanol) three times. Then, staining with 0.03% Fast Green solution in 1% acetic acid (Klinipath, BE) was performed, and after dipping three times in 1% glacial acetic acid, the samples were stained with 0.25% Safranin O solution (Sigma, DE) and washed with tap water and deionized water consecutively. For alkaline phosphatase (ALP) staining, the samples were rinsed in PBS, then incubated overnight in 1% MgCl_2_ in 100 mM Tris-maleate buffer (pH 9.2), followed by 2 h of incubation at 37 °C in BM Purple (Roche, CH). The samples were then washed in deionized water, counterstained with Fast Red solution, and rinsed in tap water.

Immunohistochemistry for the detection of human collagen type I, type II, and Indian hedgehog homolog (IHH) was performed at the same time points and on consecutive sections as the histological staining. The primary antibodies used were rabbit anti-human collagen type I (1:200; PA1-36057, Thermo Scientific, United States), rabbit anti-human collagen type II (1:20; AB761, EMD Millipore Corp., DE), and rabbit polyclonal anti-IHH antibody (1:50; ab80191, Abcam, United Kingdom) diluted in blocking buffer consisting of 5% BSA (Sigma, DE) and 0.01% Triton X-100 (Merck, DE) in TBST buffer. Antigen retrieval for the collagen staining was achieved by incubating the sections in 1 mg mL^-1^ pepsin (Sigma, DE) at pH 2 for 15 min, while Uni-Trieve solution (Innovex Biosciences, United States) was used for 30 min at 70°C for IHH. Next, endogenous peroxidase activity was quenched with 3% H_2_O_2_ (Acros Organics, NL) two times for 5 min. Blocking was performed by incubating the sections for 30 min at room temperature in blocking buffer (for IHH: 10% goat serum in TBST +0.01% Triton X-100) and directly after the primary antibody was applied for overnight incubation at 4°C. Next, another 40-min blocking step was performed before applying the secondary HRP conjugated anti-rabbit antibody (1:500; Jackson ImmunoResearch, United States) for 30 min. Peroxidase activity was visualized using 3,3′-diaminobenzidine (DAB) (Enzo, United States), and after washing with deionized water, the sections were counterstained with 1% hematoxylin solution (Merck, DE) and washed with tap water.

After staining, all sections were dehydrated in a series of ethanol dilutions (70%, 95%, and 100% v/v), dried at room temperature, and cleared with Histo-Clear before mounting with Pertex mounting medium (Histolab, SE); only the ALP stain was mounted with Fluoroshield (Sigma-Aldrich, DE). All staining was imaged using an inverted microscope (Olympus IX83).

### 2.9 Micro-CT of explants

After washing and fixing as described above, ectopic explants were scanned using a micro-CT system (Phoenix Nanotom M) to visualize and quantify mineralization. Samples were scanned using a diamond-coated tungsten target at a voltage of 60 kV and a current of 170 µA with tube mode 0, exposure time of 500 ms, frame average 1, image skip 0, 2,400 images, a 0.1-mm aluminum filter, and a resolution of 4 µm voxel size in fast scan modus. CTAn and CTVox software (Bruker micro-CT, BE) applications were used for image processing, quantification, and visualization of the mineralized matrix. Mineralization was thresholded and put through a 3D space closing (round, 2) and descpeckle (<100vox) algorithm before calculating the mineralized volume as a percentage of the total construct volume.

### 2.10 Statistical analysis

Data are represented as the mean ± standard deviation. Multiple comparisons were analyzed with two-way ANOVA, followed by multiple unpaired t-tests using Holm–Sidak’s method. The results were considered significantly different for *p*-values <0.05 (*p < 0.05, **p < 0.01, and ***p < 0.001). All analyses were performed using GraphPad Prism version 9.1.2 (GraphPad Inc., United States).

## 3 Results

### 3.1 Human periosteum-derived cells in GelMA in the serum-free differentiation medium

#### 3.1.1 Human periosteum-derived cells survive when encapsulated in the GelMA hydrogel and display a proliferation pattern similar to micromass culture

**FIGURE 1:**
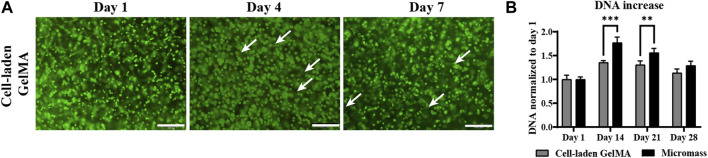
Viability and proliferation of human periosteum-derived cells encapsulated in the gelatin methacryloyl (GelMA) hydrogel cultured in a serum-free differentiation medium. **(A)** LIVE (green) and DEAD (red) staining on days 1, 4, and 7 of the *in vitro* culture. White arrows indicate dead cells. Scale bar: 200 µm. **(B)** DNA quantification of homogenized cell-laden hydrogels and control cells only in the micromass culture on days 1, 14, 21, and 28 of the *in vitro* culture. (n = 3, mean ± SD, two-way ANOVA, followed by multiple unpaired t-tests, Holm–Šídák’s multiple comparison test, *p < 0.05, **p < 0.01, and ***p < 0.001).

A LIVE/DEAD viability assay demonstrated that the vast majority of hPDCs survived the encapsulation and crosslinking procedure, and high viability was maintained up to 7 days of *in vitro* culture (Figure 1A). DNA quantification showed that the highest proliferation occurred in the first 2 weeks. Then, DNA quantity decreased toward the end of the 28-day *in vitro* culture (Figure 1B). The periosteum-derived cells cultured in micromass culture had a higher DNA quantity on day 14 and day 21; however, by the end of the culture period, this difference was insignificant.

#### 3.1.2 Human periosteum-derived cells encapsulated in GelMA produce a matrix with cartilage-like tissue components and chondrogenic markers as well as (pre-)hypertrophic tissue components and markers when cultured in a differentiation medium

**FIGURE 2:**
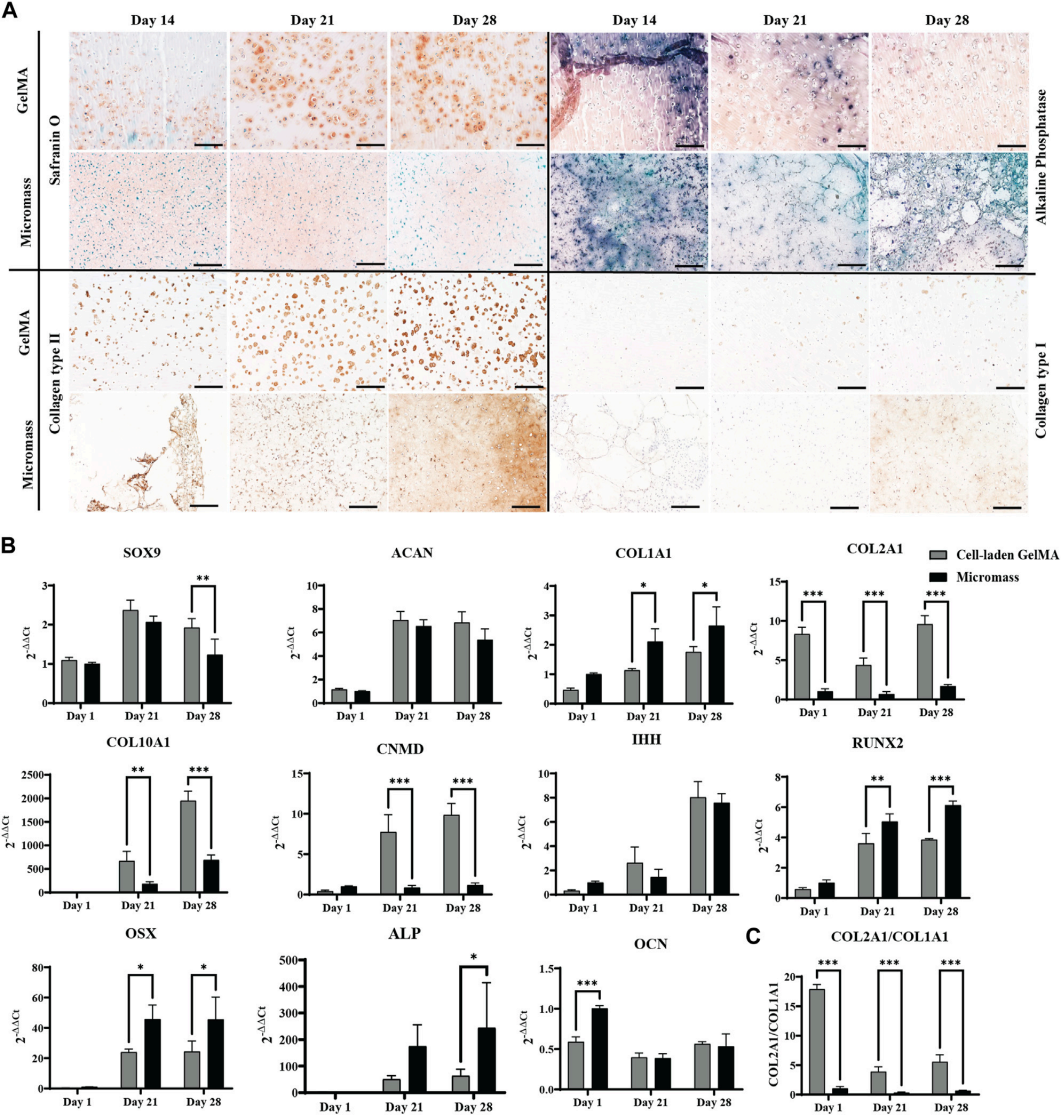
Human periosteum-derived cells encapsulated in the GelMA hydrogel at high (2.10^7^ cells mL^-1^) cell density and control micromass culture (scaffold-free, 2.10^7^ cells mL^-1^) cultured in the serum-free differentiation medium. **(A)** Safranin O and alkaline phosphatase (ALP) staining and collagen type II and type I immunohistochemistry. Scale bar: 200 µm. **(B)** Relative mRNA expression of gene markers. **(C)** Ratio of COL2A1 to COL1A1 expression. All samples are normalized to micromass culture on day 1 (n = 3, mean ± SD, multiple unpaired t-tests, Holm–Šídák’s multiple comparisons method (α = 0.05), *p < 0.05, **p < 0.01, and ***p < 0.001).

Culturing the constructs in a serum-free differentiation medium based on TGF-β1, BMP-2, and GDF-5, including low concentrations of BMP-6 and FGF-2, resulted in matrix formation. Histological staining and immunohistochemistry were performed to obtain more insights into the nature of this matrix (Figure 2A).

From day 14 onwards of the *in vitro* culture, Safranin O staining showed the presence of sulfated glycosaminoglycans (GAGs) in the cell-laden GelMA constructs, as well as in the micromasses. In the GelMA constructs, periosteum-derived cells seemed to increasingly produce more GAGs, starting from the periphery of the construct, gradually spreading inward. On the contrary, under the micromass culture condition, less positive Safranin O staining was observed by day 28. Immunohistochemistry for collagen type II indicated that the cells also produce the most abundant structural proteins found in cartilage tissue. Additionally, the matrix deposition pattern was more pericellular in cell-laden GelMA, with regions of GelMA without sulfated GAGs, as opposed to the uninterrupted matrix between the cells of the micromasses. It can be noted that a portion of the cells in GelMA has a more rounded morphology, which is typical for chondrocyte-like cells, whereas the cell morphology of hPDCs in GelMA was less rounded, suggesting that these cells move away from the chondrogenic phenotype. These findings corroborated the quantitative gene expression profile shown in Figure 2B.

Chondrogenic transcription factor SOX9 was upregulated on days 21 and 28 but displayed a significant difference between GelMA samples and micromasses on day 28. ACAN, the gene coding for aggrecan, a common proteoglycan found in GAGs, was upregulated under both conditions. The main differences between cells in the micromass and cells in GelMA were found in collagen expression. Gene expression analysis showed that hPDCs, when encapsulated in GelMA, had a 6-fold higher expression of COL2A1 and a more than 2-fold lower expression of COL1A1, a protein which is not typically found in hyaline cartilage but more so in fibrous cartilage and bone, yielding a higher chondrogenic index (COL2A1/COL1A1) for the hydrogel-based constructs. This suggests that cells in the hydrogel have a higher tendency toward the chondrogenic lineage when encapsulated in GelMA as opposed to in micromass culture. However, the most striking difference was observed in COL10A1, where the gel-embedded cells displayed significantly higher gene expression, i.e., almost 2,000-fold *versus* 700-fold on day 28. The high expression of COL10A1, especially in GelMA samples, suggested that the skeletal progenitor cells were pushed toward hypertrophy or that they had a transient chondrogenic phenotype.

The IHH gene, regulating chondrocyte hypertrophy and endochondral bone formation, was strongly upregulated by day 28 without significant differences between the conditions. This result was confirmed by immunohistochemistry (Supplementary Figure S1A). Additionally, the gene coding for chondromodulin (CNMD), associated with pre-hypertrophic cartilage, was strongly upregulated in hPDCs encapsulated in GelMA, nine times more so than the same cells in the micromass culture.

While the expression of (hypertrophic) chondrogenic markers was typically higher in GelMA-encapsulated samples than that in micromasses, the opposite was true for the osteogenic markers. For example, osteoblast differentiation transcription factor RUNX2 was observed under both conditions, although it was significantly higher in the scaffold-free micromass culture. More downstream in osteoblast differentiation, OSX or SP7 expression was expected, which was increased on day 21 under both conditions but significantly higher for micromasses than for cell-laden GelMA. ALP was observed in histology, and its presence was confirmed by upregulated gene expression, indicating hypertrophy. The expression of osteocalcin (OCN), a gene marker for bone remodeling, was significantly lower in GelMA on day 1, and with time, it also decreased under the micromass condition, showing no upregulation, which was confirmed by negative immunostaining (data not shown).

#### 3.1.3 Pre-cultured constructs of human periosteum-derived cells encapsulated in GelMA produce cartilage-like templates that undergo endochondral ossification *in vivo*

**FIGURE 3:**
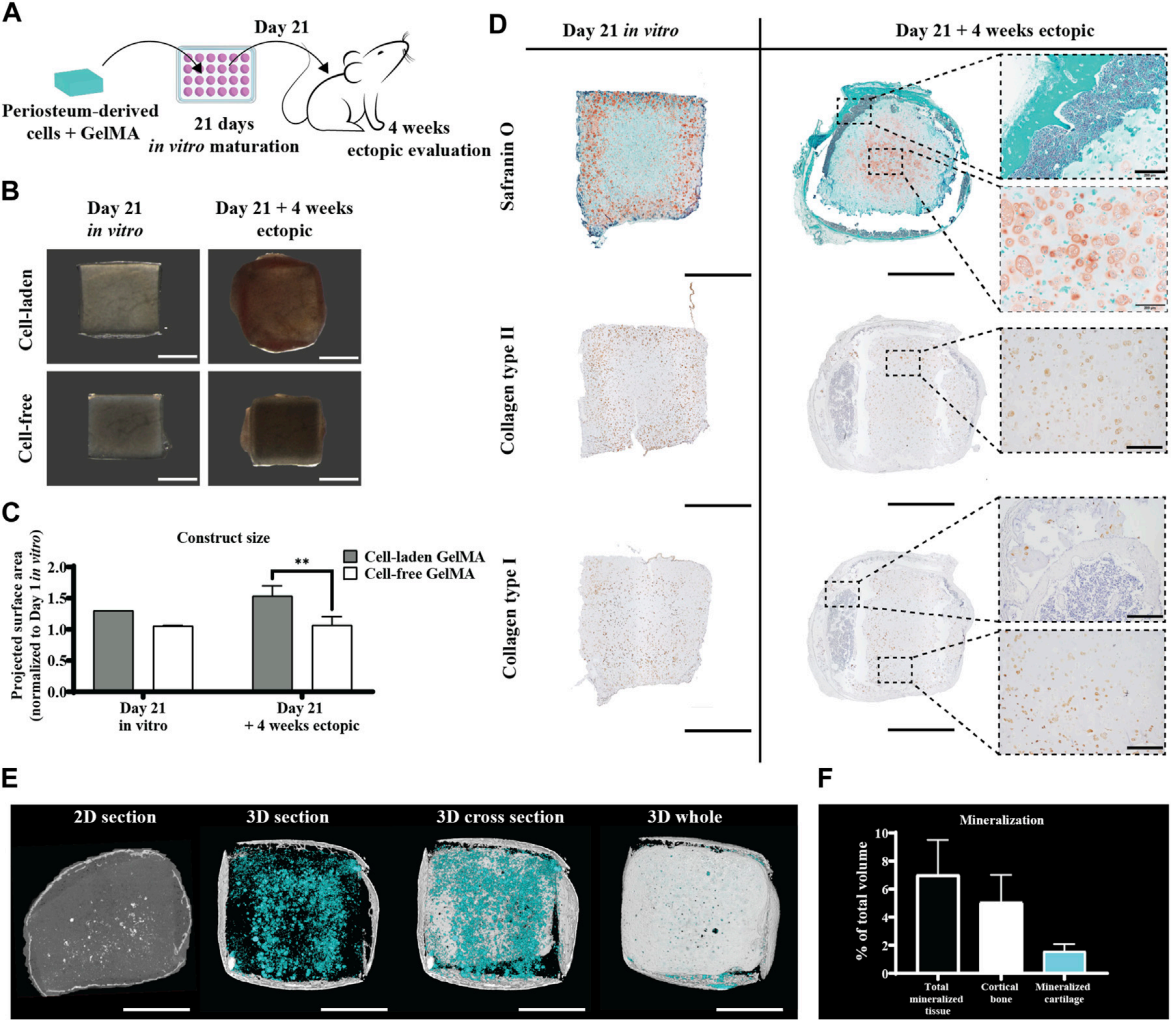
Ectopic *in vivo* evaluation of the *in vitro* differentiated human periosteum-derived cell-laden GelMA hydrogel. **(A)** Schematic diagram of the ectopic evaluation after *in vitro* differentiation. **(B)** Macroscopic appearance and **(C)** projected surface area of cell-laden and cell-free constructs before (n = 1) and after (n = 4) 4-week ectopic evaluation normalized to cell-laden constructs on day 1 of the *in vitro* culture (mean ± SD, two-way ANOVA with multiple comparisons test using Šídák’s multiple comparison test, *p < 0.05, **p < 0.01, and ***p < 0.001). Scale bar: 2 mm **(D)** Histological evaluation of cell-laden constructs before and after 4-week ectopic evaluation, including Safranin O staining and collagen type I and type II immunostaining. Scale bar: 2 mm; inset scale bar: 200 µm. **(E)** Micro-CT analysis including a 2D section of the ROI and 3D renditions of a 500-µm thick section, center cross section, and whole construct. Scale bar: 2 mm **(F)** Quantification of micro-CT analysis displaying mineralization as a % of the total construct volume (n = 4).

When hPDC-laden GelMA constructs were implanted ectopically in nude mice without an *in vitro* differentiation period, no relevant matrix deposition was observed (data not shown). However, when the formation of a cartilaginous matrix was induced by culturing the construct in a serum-free differentiation medium for 21 days (see Figure Figure3D, left panel), this matrix changed after a 4-week *in vivo* period. The periphery of the construct, which showed GAG-depositing cells *in vitro*, had turned into an osseous matrix, showing signs of cortical bone and bone marrow *in vivo* (see Figure 3D, right panel). In contrast, the inside of the construct, which did not display matrix deposition *in vitro*, developed a Safranin O-positive region including speckles of mineralization, suggesting the formation of mineralized cartilage. Correspondingly, collagen type II deposition is, overall, reduced as the collagen type II-positive outer regions are the regions undergoing ossification. However, an increase in collagen positivity was observed in the center. Collagen type I was detected only pericellularly in the *in vitro* samples, but *in vivo*, it was also observed in the osseous matrix that was formed. In the softer core of the construct, collagen type I positivity seemed to be somewhat reduced. The size of the constructs increased slightly as indicated by a 30% increase in the projected surface area compared to freshly fabricated samples after the *in vitro* culture and a 54% increase after 4 weeks *in vivo* (Figures Figure3B,C). This increase in size is cell-mediated since the cell-free GelMA constructs do not significantly change in size upon implantation. Micro-CT revealed mineralized tissue in all explants, with an average of 7% ± 2.5% of the total volume, including 5% ± 2% cortical bone on the periphery of the construct (Figures 3E,F).

### 3.2 iPSC-derived chondrocytes in GelMA in the serum-free differentiation medium

#### 3.2.1 iPSC-derived chondrocytes survive and proliferate in the serum-free differentiation medium, similar to scaffold-free culture conditions

**FIGURE 4:**
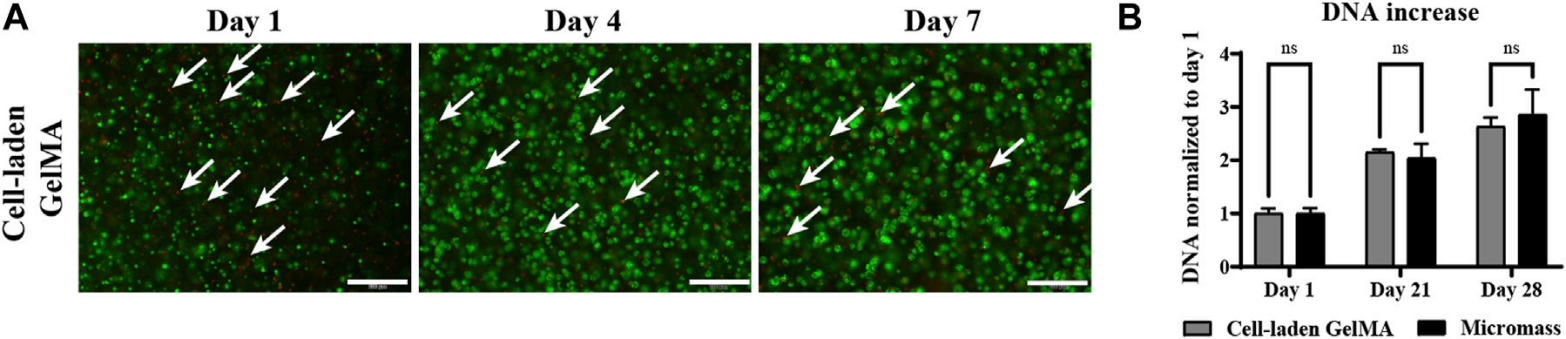
Human induced pluripotent stem cell (iPSC)-derived chondrocytes encapsulated in GelMA (2.107 cells/mL) and control cells only in the micromass culture (2.107 cells/mL) in the serum-free differentiation medium. **(A)** LIVE (green) and DEAD (red) staining on days 1 and 7 of the *in vitro* culture. White arrows indicate dead cells. Scale bar: 200 µm. **(B)** DNA quantification of homogenized cell-laden hydrogels on days 1, 21, and 28 of the *in vitro* culture (n = 3) normalized to day 1 (n = 3 mean ± SD, two-way ANOVA followed by multiple unpaired t-tests, Holm–Šídák’s multiple comparisons method (α = 0.05) ns: not significant).

A LIVE/DEAD assay showed that the majority of iPSC-derived chondrocytes survived the encapsulation and construct fabrication procedure, and that high viability was sustained over an *in vitro* culture period in a serum-free differentiation medium of 7 days (Figure 4A). DNA quantification in the homogenized constructs indicated that iPSC-derived chondrocytes cultured in GelMA proliferated similar to cells cultured in micromasses, i.e., scaffold-free conditions. On days 1, 21, and 28, no significant differences between the two construct types in DNA quantity or relative DNA increase were observed Figure 4B).

#### 3.2.2 iPSC-derived chondrocytes encapsulated in the GelMA hydrogel produce a cartilage-like matrix when cultured in the serum-free differentiation medium

**FIGURE 5:**
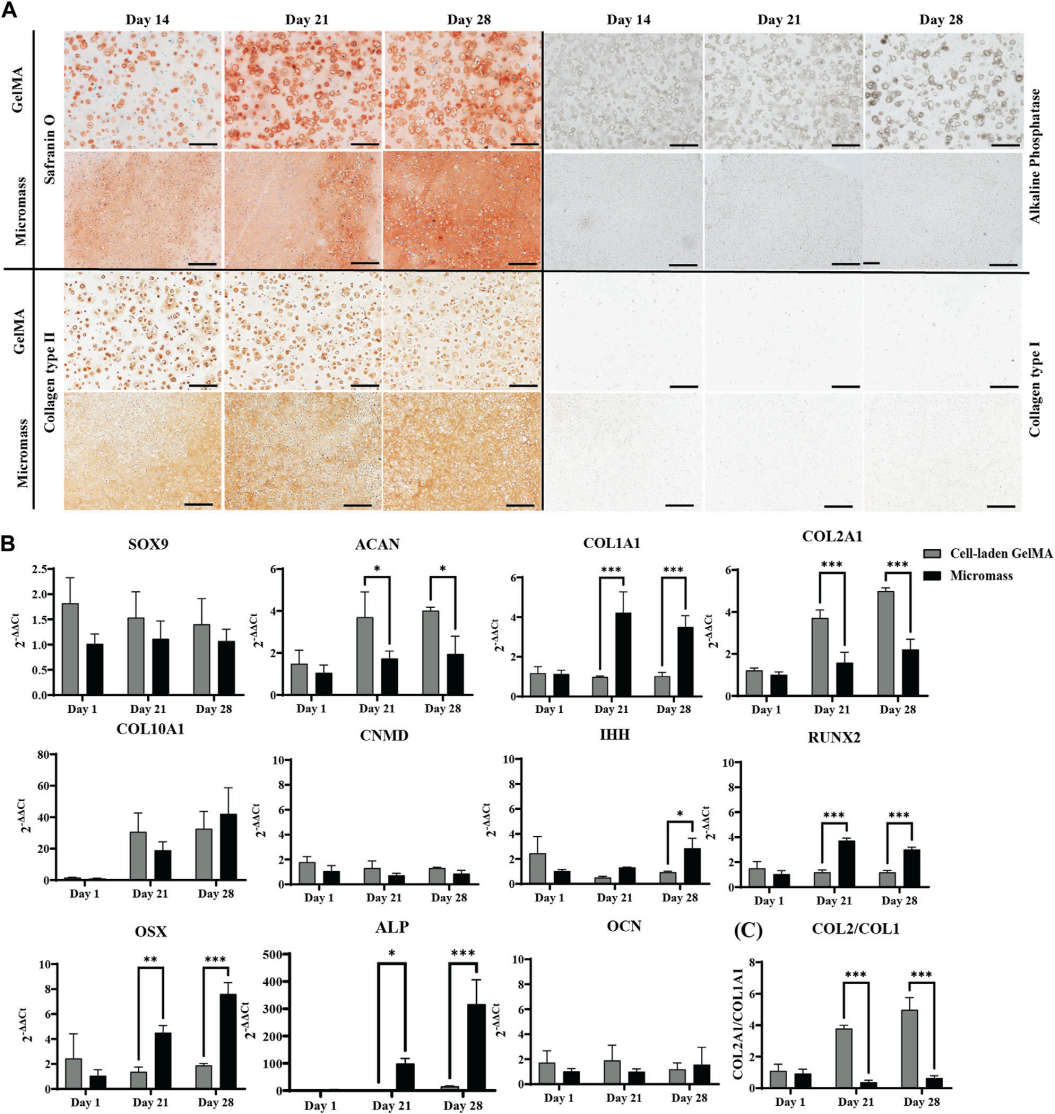
Human iPSC-derived chondrocytes encapsulated in the GelMA hydrogel at a high (2.10^7^ cells mL^-1^) cell density and control micromass culture (scaffold-free, 2.10^7^ cells mL^-1^) cultured in a serum-free differentiation medium. **(A)** Safranin O and ALP staining and collagen type II and type I immunohistochemistry. Scale bar: 200 µm. **(B)** Relative mRNA expression of gene markers. **(C)** Ratio of COL2A1 to COL1A1 expression. All samples are normalized to micromass culture on day 1 (n = 3, mean ± SD, multiple unpaired t-tests, Holm–Šídák’s multiple comparisons method (α = 0.05), *p < 0.05, **p < 0.01, and ***p < 0.001).

Since the GelMA hydrogel itself does not stain (see Supplementary Figure S1B), the intense pericellular Safranin O staining observed by day 14 of the *in vitro* culture indicates GAG deposition by the cells. At this time point, a considerable number of cells that do not produce GAGs were also present. From day 21 onward, Safranin O staining was more intense and also more dispersed throughout the gel matrix. A similar pattern was observed in immunostaining for collagen type II. Collagen type I, which is typical to fibrous cartilage and bone, is not detected in GelMA-based samples. In micromasses, however, a limited amount of collagen type I was observed at the end of the *in vitro* culture period in the periphery of the sample (Supplementary Figure S2A). Gene expression analysis (Figure 5B) showed no significant differences in SOX9 expression; however, significantly increased ACAN, coding for the proteoglycans necessary to form GAGs, was expressed in iPSC-derived chondrocytes encapsulated in GelMA compared to scaffold-free micromasses. The same was true for COL2A1, with a highly significant difference between the two construct types. For COL10A1, no significant differences were observed. COL1A1 was not upregulated in cells encapsulated in GelMA, as opposed to the scaffold-free control. This led to a significantly higher differentiation index, i.e., the ratio of COL2A1 to COL1A1, in the cell-laden gels than that in micromass controls (Figure 5C).

By encapsulating iPSC-derived chondrocytes in the GelMA hydrogel, we aim to obtain a tissue engineered cartilage construct. Nevertheless, for the sake of completeness, markers for (pre)hypertrophy were also examined. CNMD, IHH, RUNX2, OSX, and OCN were not detected in the cell-laden GelMA constructs. At the end of the 28-day *in vitro* culture, mild upregulation of ALP was observed; however, this was not translated on the protein level, as demonstrated by ALP staining. For the micromasses, we could observe the upregulation of pre-hypertrophic and osteogenic genes IHH, RUNX2, OSX, and ALP, suggesting transdifferentiation of the iPSC-derived chondrocytes in the absence of the supporting GelMA hydrogel.

#### 3.2.3 Pre-cultured constructs of iPSC-derived chondrocytes encapsulated in GelMA form large cartilaginous tissue mimics *in vivo*

**FIGURE 6:**
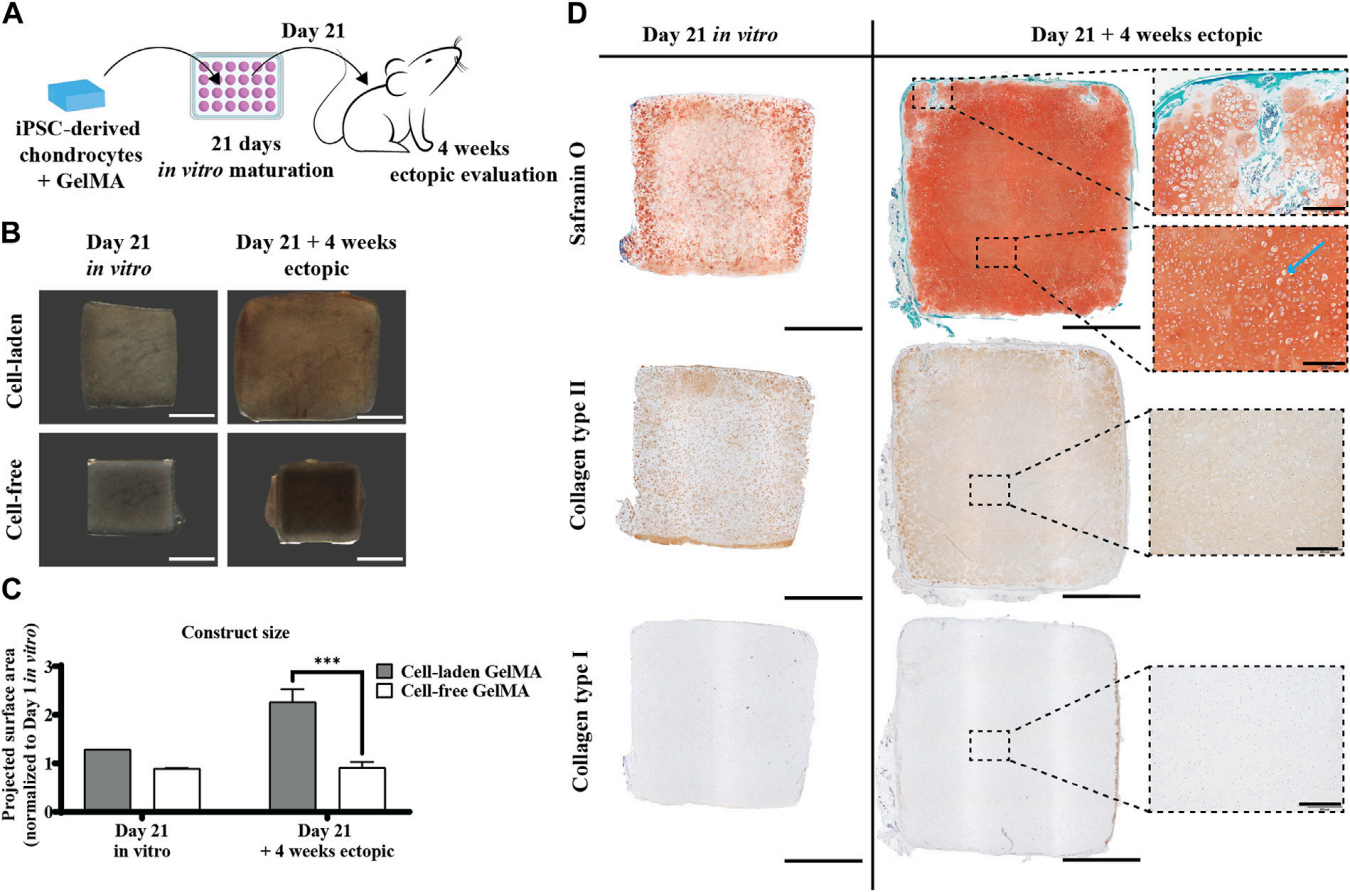
Ectopic *in vivo* evaluation of the *in vitro* differentiated human iPSC-derived chondrocyte-laden GelMA hydrogel. **(A)** Schematic diagram of the ectopic evaluation after *in vitro* differentiation. **(B)** Macroscopic appearance and **(C)** projected surface area of cell-laden and cell-free constructs before (n = 1) and after (n = 4) 4-week ectopic evaluation normalized to cell-laden constructs on day 1 of the *in vitro* culture (mean ± SD, two-way ANOVA with multiple comparisons test using Šídák’s multiple comparison test, *p < 0.05, **p < 0.01, and ***p < 0.001). Scale bar: 2 mm. **(D)** Histological evaluation of cell-laden constructs before and after 4-week ectopic evaluation, including Safranin O staining and collagen type I and type II immunostaining. Scale bar: 2 mm; inset scale bar: 200 µm.

To assess the necessity of an *in vitro* matrix formation period for *in vivo* cartilage formation, constructs with and without pre-culture implanted ectopically in iPSC-derived chondrocyte-laden GelMA constructs that were cultured in a serum-free differentiation medium developed a Safranin O-positive matrix, with an abundant presence of collagen type II (Figure 6D, left panel). When these constructs were implanted subcutaneously in nude mice, an increase in size, together with a maturation of the matrix, was observed. Construct sizes increased by 29% after the *in vitro* culture, but after *in vivo* evaluation, their size had increased to 226% of the original size after fabrication (Figures 6B,C). Together with this increase in size, an intensification of the Safranin O staining was observed, leaving no more space between the pericellular neotissue, as opposed to what was observed *in vitro*. In the periphery of the construct, some Safranin O-negative spots were observed, where, inside a region with a hypertrophic phenotype, some mineralization was observed (Supplementary Figure S4A,B). In addition, the typical chondrocyte lacunae (blue arrow) found in mature cartilage were apparent. Additionally, the collagen type II network had densified and dissipated throughout the matrix, as opposed to the intense pericellular staining observed *in vitro*. Deposition of collagen type I was not observed (Figure 6D, right panel).

#### 3.2.4 iPSC-derived chondrocytes encapsulated in GelMA produce a cartilage-like matrix *in vivo*, even without an *in vitro* matrix formation period

**FIGURE 7:**
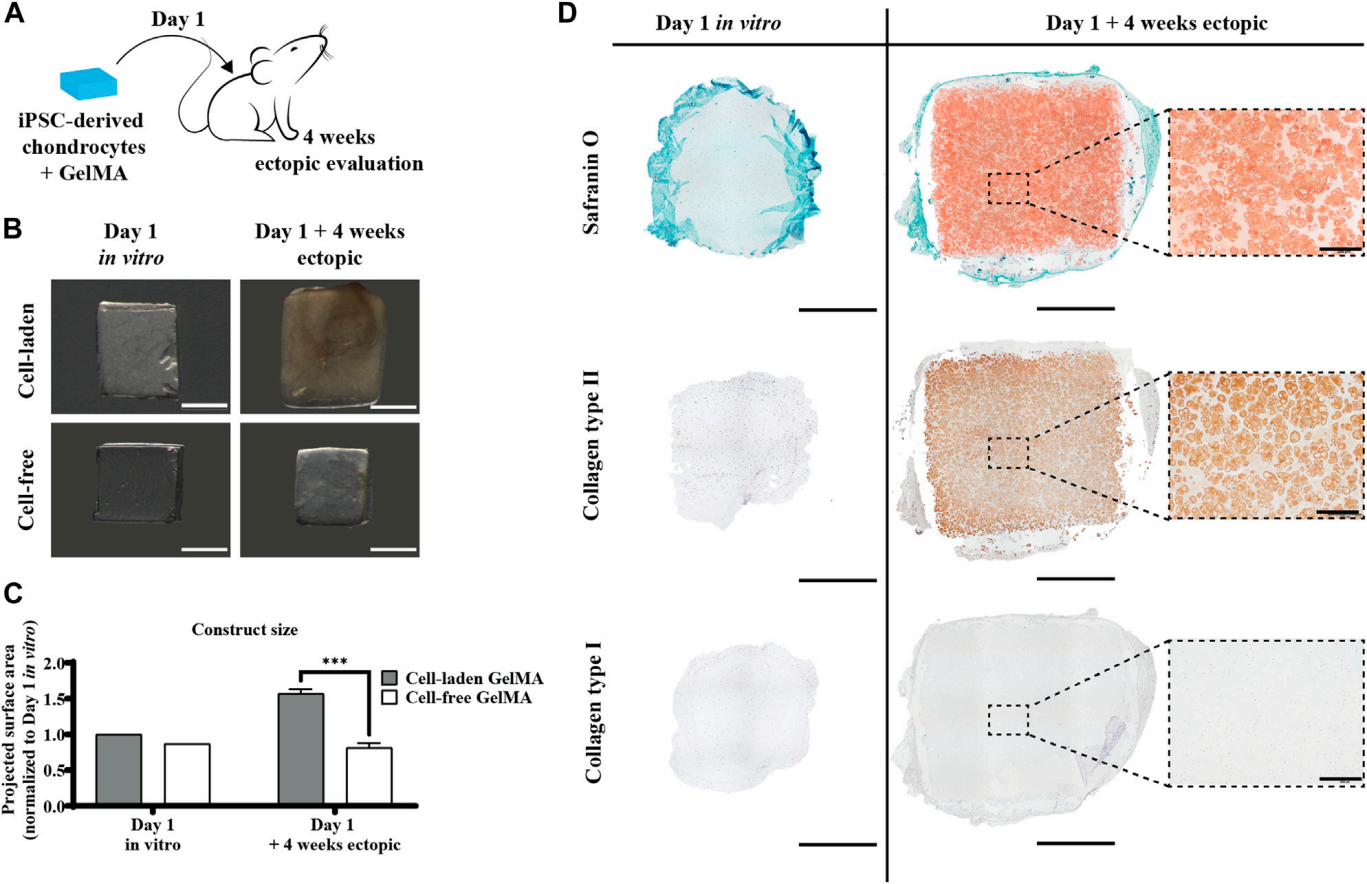
Direct ectopic *in vivo* evaluation of the human iPSC-derived chondrocyte-laden GelMA hydrogel. **(A)** Schematic diagram of direct ectopic evaluation. **(B)** Macroscopic appearance and **(C)** projected surface area of cell-laden and cell-free constructs before (n = 1) and after (n = 4) 4-week ectopic evaluation normalized to cell-laden constructs on day 1 of the *in vitro* culture (mean ± SD, two-way ANOVA with multiple comparisons using Šídák’s multiple comparison test, *p < 0.05, **p < 0.01, and ***p < 0.001). Scale bar: 2 mm. **(D)** Histological evaluation of cell-laden constructs before and after 4-week ectopic evaluation, including Safranin O staining and collagen type I and type II immunostaining. Scale bar: 2 mm; inset scale bar: 200 µm.

To assess the inherent *in vivo* matrix forming capacity of the iPSC-derived chondrocyte-laden constructs, they were also implanted ectopically without a prior *in vitro* culture period, i.e., when no matrix was formed yet (Figure 7D, left panel). Interestingly, these constructs developed a safranin O- and collagen type II-positive matrix after 4 weeks *in vivo* (Figure 7D, right panel). In contrast to pre-cultured constructs, there was a certain amount of GelMA left (lighter zones) between the newly formed matrix. The matrix deposition pattern was more similar to the *in vitro* situation, with intense type II collagen focused around the cells. Furthermore, here, no collagen type I was detected. When implanting the constructs without prior *in vitro* matrix formation, no mineralization was detected after 4 weeks *in vivo* (Supplementary Figure S4). The construct size after *in vivo* implantation had increased by 29% (Figures 7B,C).

## 4 Discussion

In this study, we show that the encapsulation of human periosteum-derived cells in GelMA hydrogels yields viable tissue constructs that form a cartilage-like matrix *in vitro*, which undergoes endochondral ossification *in vivo*. This follows the paradigm of developmental engineering, where the formation of an intermediate tissue, the cartilaginous template, is followed by ossification *in vivo*, hereby recapitulating the body mechanisms for bone tissue development and fracture healing (Lenas et al., 2009). Moreover, the implantation of a softer cartilaginous tissue intermediate, as opposed to mature bone tissue, could encourage lateral integration with the host tissue in an orthotopic setting (Mendes et al., 2020). It has previously been shown in the literature that MSCs can mimic endochondral ossification (Visser et al., 2015a; Confalonieri et al., 2018). However, in scaffold-free approaches, there are challenges related to upscaling the aggregation processes and the size of the tissue engineered constructs. Including a biomimetic biomaterial like GelMA allows matrix formation by MSCs while facilitating upscaling and shaping the implants (Yang et al., 2017a). Periosteum-derived cells appear as an interesting cell source since they are highly proliferative and have environmental-niche memory surrounding bone tissue and being the prime source for progenitor cells in the case of fracture healing (Roberts et al., 2015). Interestingly, periosteum-derived cells show chondrogenic potential even when harvested from older patients (Jansen et al., 2008).

We observed cell survival and relevant matrix production by hPDCs that are encapsulated in GelMA. Including a hydrogel as a scaffold to allow geometrical scaling up is the key, and GelMA was chosen here since it is subject to cell-mediated degradation, allows differentiation and maturation of multiple cell types including chondrocytes, MSCs, and endothelial cells, and has its application in the field of automated fabrication (Daly et al., 2018; De Moor et al., 2020; De Moor et al., 2021). The use of a biomimetic and degradable material that provides support for the cells can not only lower the initial amount of cells needed but help in controlling cell morphology and differentiation, enhancing extracellular matrix production, as indeed observed qualitatively and quantitatively in the present study (Spiller et al., 2011; Yue et al., 2015a; Yang et al., 2017b). The good biocompatibility of GelMA and the potential of MSCs from various sources to produce the matrix when embedded in GelMA have been shown by several groups (Levett et al., 2014; Visser et al., 2015a; Yue et al., 2015b; Daly et al., 2016a; Levato et al., 2017; De Moor et al., 2020). In addition, the tissue engineering potential of periosteum-derived cells has been demonstrated in scaffold-free approaches such as micromass and spheroid culture (Mendes et al., 2018; Nilsson Hall et al., 2020). When culturing GelMA-embedded hPDCs *in vitro* for up to 28 days in TGF-β1, BMP-2, and GDF-5-based differentiation medium, we observed the upregulation of chondrogenic markers such as SOX9, ACAN, and COL2A1. In addition, hypertrophic markers like COL10A1, IHH, and CNMD were also upregulated. Previously, the presence of hypertrophic markers in *in vitro* cultured MSCs in GelMA has been demonstrated as MSCs typically differentiate beyond the chondrocyte phenotype (Levato et al., 2017; Daly et al., 2018; Luo et al., 2020). When implanted *in vivo*, ectopic mineralization of the constructs was observed. The behavior of periosteum-derived cells in micromass culture has been evaluated previously, and the findings were that ectopically, either bone ossicles or mineralized cartilage was formed (Mendes et al., 2018). Our cell-laden GelMA constructs that, prior to implantation in nude mice, had been cultured *in vitro* for 21 days generated tissue constructs with marrow compartments and mineralization in the center of the construct, as well as a cortical bone-like appearance in the outer region, as shown by histology and micro-CT. The disparity between the inner and outer regions could be explained by differences in the exposure to blood vessels and, thus, exposure to oxygen. Visser et al. observed endochondral bone formation by equine BM-MSCs after 8 weeks in the subcutaneous pockets of rats after a 2-week *in vitro* pre-culture with TGF-β2 based-medium (Visser et al., 2015a). Furthermore, here, the cortical bone was formed in the outer region of the construct, while softer tissue was found in the center. Daley et al. showed the mineralization of 4-week chondrogenically (TGF-β3) primed porcine MSCs encapsulated in GelMA, with a mineralized volume of approximately 5% after 4 weeks *in vivo*, which is slightly lower than the 7% reported here (Daly et al., 2016b). Our data confirm these findings while using a human cell source, which is more accessible and proliferative than BM-MSCs (Roberts et al., 2015).

In the same setup, the performance of chondrocytes was tested, with the aim of generating a stable cartilage top layer to be used in combination with hPDC-laden GelMA for osteochondral regeneration. *In vitro*, a typical articular cartilage-like matrix formation by the iPSC-derived chondrocytes was observed histologically and confirmed by gene expression analysis. The abundant deposition of GAGs and collagen type II, in the absence of collagen type I, surpasses the quality of matrix production that has previously been shown by equine and human primary chondrocytes encapsulated in GelMA (Boere et al., 2014; Visser et al., 2015b; Mouser et al., 2016; Hölzl et al., 2022). This difference in metabolic performance could be attributed to the age of the chondrocytes used in each study as older primary cells tend to produce a less functional matrix (Loeser, 2009; Lee et al., 2015; Castro-Viñuelas et al., 2020). In corroboration with our observation that more matrix is deposited in our setup than with primary chondrocytes, we also obtained a higher compressive modulus (250 kPa; see Supplementary Figure S3) in a shorter *in vitro* culture period than previously reported, which is supported by the fact that the compression modulus is correlated with the GAG content of tissue engineered constructs (Pfeiffer et al., 2008; Levato et al., 2017). Since the unconfined compression modulus of native articular cartilage ranges from 240 to 900 kPa, our *in vitro* cultured samples lie at the lower end, which corresponds to the mechanical properties of immature cartilage (Mow and Guo, 2002; Little et al., 2011; Beck et al., 2016). Since chondrocytes are highly mechano-sensitive cells, the mechanical properties of the tissue engineered constructs could be improved by mechanical stimulation in the *in vitro* phase or even by the natural stimuli in an orthotopic *in vivo* setting (Steinmetz et al., 2015; Anderson and Johnstone, 2017; Cai et al., 2021; Statham et al., 2021). Interestingly, the initial modulus of approximately 25 kPa in uncultured samples (see Supplementary Figure S3) closely resembles the modulus of the perichondral space, which guides chondrocytes in their matrix production (Bachmann et al., 2020). Possibly, part of the explanation for the difference in phenotype and matrix formation between gel-based and scaffold-free constructs can be found here.

When transferring our *in vitro* formed tissue constructs to an *in vivo* ectopic setting, we observed the maturation of the tissue, as apparent by the increased amount of matrix formed, and the decrease in the gel material. We noticed that there are very few ectopic studies with GelMA in a cartilage context, but, for example, Boere et al. showed the implantation of reinforced chondrocyte-laden GelMA after 2 weeks *in vitro* and observed less GAG formation after 8 weeks *in vivo* compared to 6 weeks *in vitro* (Boere et al., 2014). In our setup, small indications of mineralization were observed on the edges of the explants after 4 weeks in the ectopic assay. We did not assess whether further mineralization occurs with a longer ectopic *in vivo* period; however, based on the literature, this is not expected in a mechanically functional environment (Cai et al., 2021; Richardson et al., 2021; Tam et al., 2021). However, orthotopic experiments with longer term evaluation periods will need to be conducted to confirm this. We also evaluated the ectopic potential of naïve cell-laden constructs, i.e., without *in vitro* pre-culture, and observed an abundant Safranin O-positive and collagen type II containing matrix, albeit less mature than in the pre-cultured samples. No mineralization was observed here. The iPSC-derived chondrocytes in our setup appeared more potent than that observed in primary articular chondrocytes, for example, Li et al. showed only limited matrix formation by unprimed bovine articular chondrocytes after a longer period of 6 weeks in the subcutaneous dorsal pockets of nude mice (Li et al., 2017). From a translational perspective, a shorter *in vitro* culture period would greatly decrease the complexity and cost of fabricating a tissue engineered implant. Although a softer and more immature cartilage matrix could be advantageous in terms of integration with the host tissue, the benefits of a shorter *in vitro* culture period should be weighed up against the decreased maturity or slower maturation of the construct *in vivo* (Moretti et al., 2005; Miot et al., 2012; Mendes et al., 2020; Vázquez et al., 2021). These differences need to be evaluated in an orthotopic setting. Taken together, iPSC-derived chondrocytes, as well as periosteum-derived cells, are highly accessible sources of potent tissue-forming cells that, in combination with the GelMA hydrogel, form a promising strategy for osteochondral tissue engineering.

## 5 Conclusion

Taken together, we showed that GelMA supports human periosteum-derived cells in the formation of cartilaginous (pre)-hypertrophic tissue *in vitro* while guiding endochondral ossification in an ectopic setting. Additionally, iPSC-derived chondrocytes encapsulated in GelMA produce an extracellular matrix that corresponds to immature cartilage *in vitro*, which undergoes further maturation *in vivo*. We qualitatively and quantitatively demonstrated that cell-laden GelMA constructs biologically outperformed their cell-only counterparts in *in vitro* studies. In our *in vivo* studies, we generated cartilage, mineralized cartilage, and cortical bone using these two cell types encapsulated in the same GelMA hydrogel and using the same serum-free medium. The two biological inks form a powerful combination for an automated multi-component osteochondral tissue engineering approach. Future work should combine these two cell-laden gels in a bioprinting setup, as well as test the performance of both bioinks in an orthotopic setting, providing an adequate biological and mechanical environment.

## Data Availability

The original contributions presented in the study are included in the article/Supplementary Material; further inquiries can be directed to the corresponding author.
