# Supplementary material for: In vitro and in vivo evaluation of periosteum-derived cells and iPSC-derived chondrocytes encapsulated in GelMA for osteochondral tissue engineering
Source: Front Bioeng Biotechnol. 2024 Apr 11;12:1386692. doi: 10.3389/fbioe.2024.1386692 (PMC11043557; doi:10.3389/fbioe.2024.1386692)
Supplement: Supplementary file 1 [file Image1.PDF]

Supplementary Material

1 Supplementary Figures

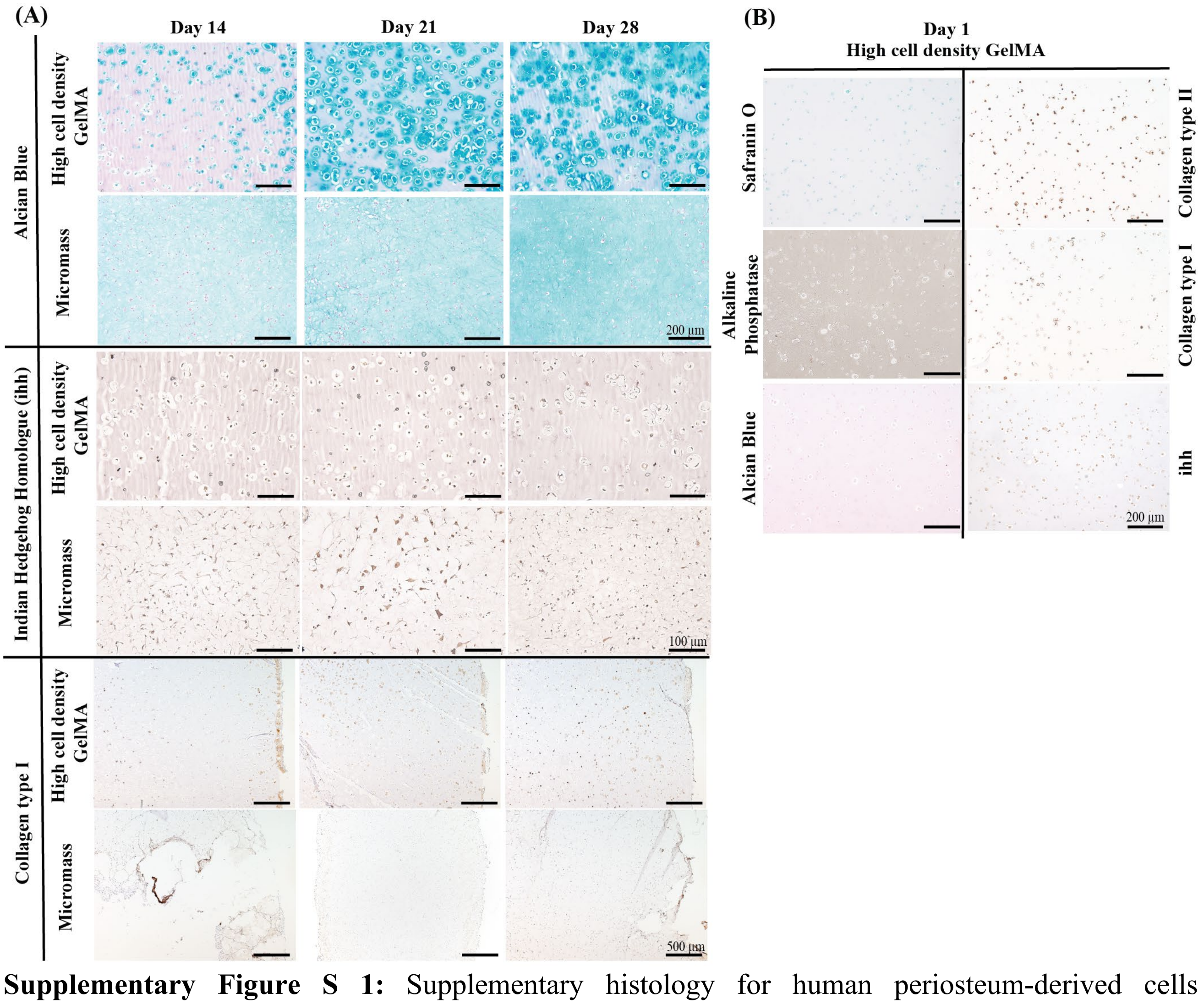

Supplementary Figure S 1: Supplementary histology for human periosteum-derived cells encapsulated in GelMA hydrogel at high (2.107 cells mL-1) cell density and control micromass culture (scaffold -free, 2.107 cells mL-1) cultured in serum-free differentiation medium. (A) Alcian Blue staining and indian hedgehog homologue (ihh) and collagen type I immunohistochemistry. Scale bar Alcian Blue: 200 µm, scale bar ihh: 100 µm, scale bar Collagen type I: 500 µm. (B) Day 1 staining of cell-laden GelMA for Safranin O, Collagen type II, Alkaline Phosphatase and Collagen type I. Scale bar: 200 µm.

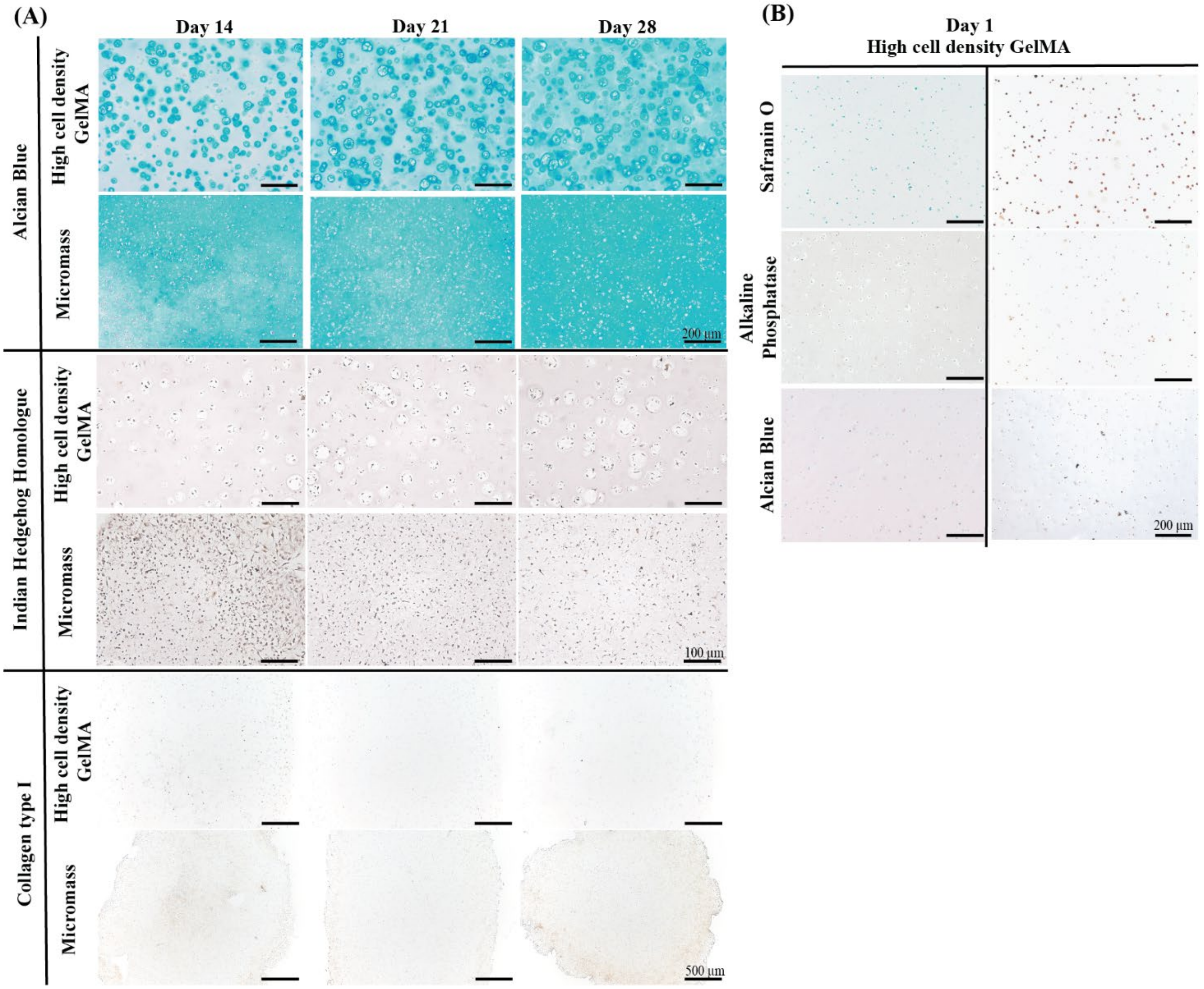

Supplementary Figure S 2: Supplementary histology for human iPSC-derived chondrocytes encapsulated in GelMA hydrogel at high (2.107 cells mL-1) cell density and control micromass culture (scaffold -free, 2.107 cells mL-1) cultured in serum-free differentiation medium. (A) Alcian Blue staining and indian hedgehog homologue (ihh) and collagen type I immunohistochemistry. Scale bar Alcian Blue: 200 µm, scale bar ihh: 100 µm, scale bar Collagen type I: 500 µm. (B) Day 1 staining of cell-laden GelMA for Safranin O, Collagen type II, Alkaline Phosphatase and Collagen type I. Scale bar: 200 µm.

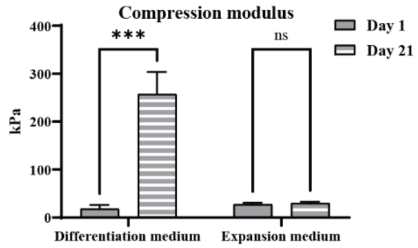

Supplementary Figure S 3: Compression modulus of iPSC-derived chondrocyte-laden GelMA constructs (n=10) on day 1 versus day 21 of in vitro culture, with iPSC-derived chondrocyte-laden GelMA cultured in expansion medium as controls (n=5). Unconfined vertical uniaxial compression experiments performed using a LM1 Testbench (TA Instruments, USA) with 2.5 N load cell. Cell-laden constructs were compressed at a rate of 10%/s, until a compression of 20 % of the thickness of the sample was reached, as suggested by Elahi et al.[68] The compression modulus was calculated as the slope of the stress/strain curve in the 10-15% strain range. (mean ± SD, 2-way ANOVA followed by multiple unpaired t-tests, Holm-Šídák's multiple comparisons method (α=0.05) ***p < 0.001, ns = not significant)

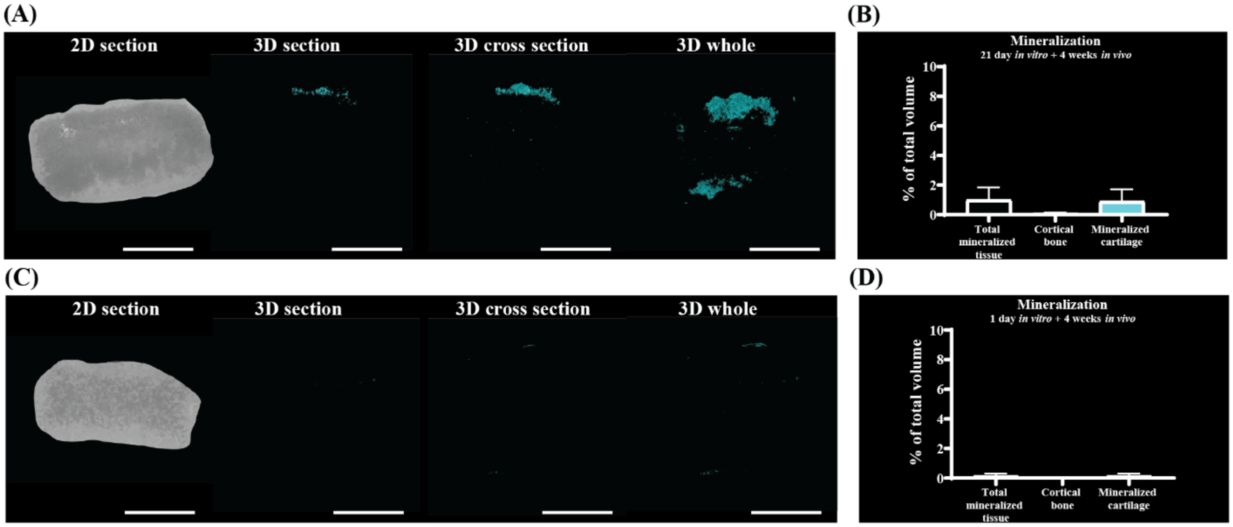

Supplementary Figure S 4: Ectopic in vivo evaluation of in vitro differentiated human iPSC-derived chondrocyte-laden GelMA hydrogel after 21 (A-B) or 1 (C-D) days in vitro. (A) MicroCT analysis including a 2D section of the ROI and 3D renditions of 500 µm thickness section, center cross section and whole construct. Scale bar: 2 mm (F) Quantification of MicroCT analysis displaying mineralization as a % of total construct volume (n=4).
